# Protocol for a randomised controlled trial evaluating the effect of a CBT-I smartphone application (Sleep Ninja®) on insomnia symptoms in children

**DOI:** 10.1186/s12888-023-05185-x

**Published:** 2023-09-20

**Authors:** M. Subotic-Kerry, A. Werner-Seidler, B. Corkish, P. J. Batterham, G. Sicouri, J. Hudson, H. Christensen, B. O’Dea, S. H. Li

**Affiliations:** 1 Black Dog Institute, University of New South Wales, Sydney, NSW Australia; 2 Black Dog Institute and School of Psychology, University of New South Wales, Sydney, NSW Australia; 3 Centre for Mental Health Research, Australian National University, Canberra, Australia; 4 Faculty of Medicine and Health, University of New South Wales, Sydney, NSW Australia

**Keywords:** Insomnia, Depression, Anxiety, Cognitive behaviour therapy, Children, Mobile application

## Abstract

**Background:**

Sleep is necessary for healthy development and mental wellbeing. Despite this, many children do not get the recommended duration of sleep each night, and many experience sleep problems. Although treatable, existing interventions for sleep disturbance are time-consuming, burdensome for families, and focus on providing behavioural strategies to parents rather than upskilling children directly. To address this gap, we modified Sleep Ninja®, an evidence-based cognitive behavioural therapy for insomnia (CBT-I) smartphone app for adolescent sleep disturbance, to be appropriate for 10 to 12 year olds. Here, we describe the protocol for a randomised controlled trial to evaluate the effect of Sleep Ninja on insomnia and other outcomes, including depression, anxiety, sleep quality, and daytime sleepiness, and explore effects on the emergence of Major Depressive Disorder (MDD), compared to an active control group.

**Methods:**

We aim to recruit 214 children aged 10 to 12 years old experiencing disturbed sleep. Participants will be screened for inclusion, complete the baseline assessment, and then be randomly allocated to receive Sleep Ninja, or digital psychoeducation flyers (active control) for 6-weeks. The primary outcome, insomnia symptoms, along with depression, anxiety, sleep quality, and daytime sleepiness will be assessed at 6-weeks (primary endpoint), 3-months, and 9-months post-baseline (secondary and tertiary endpoints, respectively). A mixed model repeated measures analytic approach will be used to conduct intention-to-treat analyses to determine whether reductions in insomnia and secondary outcomes are greater for those receiving Sleep Ninja relative to the control condition at the primary and secondary endpoints. The difference in relative risk for MDD onset will be explored at 9-months and compared between conditions.

**Discussion:**

This is the first clinical trial examining the effects of a CBT-I smartphone app in children experiencing sleep disturbance. Results will provide empirical evidence about the effects of Sleep Ninja on insomnia and other mental health outcomes.

**Trial registration:**

Australian New Zealand Clinical Trials Registry (ACTRN12623000587606).

**Universal Trial Number:**

U1111-1294-4167.

**Supplementary Information:**

The online version contains supplementary material available at 10.1186/s12888-023-05185-x.

## Introduction

Sleep affects every system in the body. It is a foundational requirement for healthy social, emotional, and physical development [1]. Insufficient and disturbed sleep is associated with reduced physical health, poor academic performance, and emotional dysregulation in children [2–6]. Many children (> 30%) fail to sleep for the recommended 9–11 hours each night, and approximately 40% will experience significant sleep problems by the time they enter secondary school [7]. Like adults and adolescents, sleep problems in children are treatable with cognitive behavioural sleep interventions (collectively termed cognitive behavioural therapy for insomnia; CBT-I) [8, 9]. However, CBT-I for children is currently time-consuming, burdensome on families, and typically requires significant contribution from expert clinicians [9]. It also focuses on providing behavioural strategies to parents, rather than directly equipping children with strategies to improve their sleep [10]. Individual skill development is particularly important for children who are implementing bedtime routines independently. Research shows that this tends to happen around late primary school age (e.g., 10 to 12 years), which also coincides with parents becoming increasingly unaware of their child’s sleep problems [11].

Sleep and mental health outcomes are closely associated. For example, in adolescents, poor sleep is a risk factor for depression onset [12] and predicts anxiety symptom severity [13]. Furthermore, treatments that improve sleep also improve depression and anxiety in adults and adolescents [14–19]. These findings suggest that improving sleep in children has the potential to indirectly target other mental health symptoms, including depression and anxiety. However, the effect of sleep treatments, such as CBT-I, on depression and anxiety has not yet been adequately investigated in children.

To overcome the lack of accessible CBT-I for older children, our research team has adapted the Sleep Ninja smartphone application [20] for use among children aged 10 to 12 years old. The Black Dog Institute originally developed Sleep Ninja, a free, fully automated CBT-I smartphone application designed in consultation with adolescents and their parents [21], for adolescents aged 12 to 16 years. Sleep Ninja contains six brief lessons or ‘training sessions’ (each up to ten minutes in duration), that deliver core components of CBT-I, including psychoeducation, stimulus control, sleep hygiene, and sleep-focused cognitive therapy. It uses gaming elements to enhance user engagement, which are based on a Ninja analogy with users ‘levelling up’ on completion of a training session until they master sleep skills and become a black belt in sleep.

Previous research shows Sleep Ninja improves insomnia and reduces depression symptoms in adolescents relative to an active control, with superior effects for younger teenagers [17, 22]. Given its gamified features and readability level (i.e., for readers at the Year 5 to 6 level, aged 10 to 12 year old) of the original Sleep Ninja app, and children’s increased capacity to use interactive smartphone applications [23], Sleep Ninja required only minor modifications for adaption for 10 to 12 year olds. The key modifications included involving parents in the intervention by developing a website containing resources for parents, guardians and carers (henceforth referred to as parents) to support their child’s autonomous use of Sleep Ninja, and ensuring the suggested bedtimes were based on age-appropriate sleep duration recommendations [24]. A clinical trial is now needed to determine the effectiveness of Sleep Ninja for improving insomnia symptoms and other mental health symptoms in primary school children aged 10 to 12 years.

### Trial objectives

This protocol is reported following SPIRIT guidelines [25] to facilitate the reporting of the trial results using CONSORT guidelines [25]. The primary aim of the trial is to evaluate the effectiveness of Sleep Ninja, a CBT-I smartphone application, in reducing insomnia symptoms in children aged 10 to 12 years experiencing sleep disturbance. Using a two-arm randomised controlled trial, Sleep Ninja will be compared to an active control condition to determine the effectiveness of the app for improving insomnia symptoms after 6 weeks of use (primary endpoint), at 3-months follow-up (secondary endpoint), and at 9-months follow-up (tertiary endpoint). Secondary outcomes will be evaluated which include symptoms of depression and anxiety as well as sleep quality and daytime sleepiness, relative to the control condition. Whether reductions in depressive symptoms are mediated by improvements in sleep will be investigated, and the potential mediating role of cognitive processes, including repetitive thinking, dysfunctional beliefs about sleep and pre-sleep arousal, on treatment effects will be explored. The emergence of new cases of MDD at the tertiary endpoint will also be explored.

### Hypotheses

The primary hypothesis is that relative to the active control condition, participants who receive the Sleep Ninja app will show greater improvements in child-reported insomnia symptoms at 6-weeks post-baseline (primary endpoint). Secondary hypotheses include that relative to the control condition, participants who receive the Sleep Ninja app will show a greater reduction in symptoms of depression and anxiety, daytime sleepiness, and improved sleep quality at 6-weeks post-baseline (primary endpoint), and improvements in primary and secondary outcomes will be maintained at 3-months post-baseline. In line with previous studies, it is hypothesised that reductions in depressive symptoms will be mediated by improvements in insomnia symptoms. The number of MDD cases at 9-months post-baseline in both groups (tertiary endpoint) will be explored.

## Methods/design

### Trial design

A two-arm randomised controlled trial (RCT) with an equal allocation ratio will compare Sleep Ninja to an active control group. Outcomes include both parent- and child-reported measures, which will be assessed at baseline, post-intervention (primary endpoint, measured at 6-weeks post-baseline), three-month follow-up (secondary endpoint, measured at 3-months post-baseline), and 9-month follow-up (tertiary endpoint, measured at 9-months post-baseline). The University of New South Wales is the sponsor of this clinical trial and ethics approval was provided by the University of New South Wales Human Research Ethics Committee (HC#230070). This trial was prospectively registered with the Australian New Zealand Clinical Trials Registry on the 30th of May 2023 (ACTRN12623000587606) and has been allocated the Universal Trial Number U1111-1294-4167.

### Setting

The trial will be conducted entirely online. Data will be collected from individuals residing in Australia using the Qualtrics survey platform.

### Participants

Eligible participants are those aged 10 to 12 years old and not yet in secondary school; located in Australia; who own or have access to a smartphone; have access to the Internet, an active email address and mobile phone number; who have age-appropriate English proficiency; and who can obtain their parent’s consent. Eligible participants are also required to be experiencing sleep disturbance, as determined by a total score of ≥ 2 (i.e., indicating 2–3 nights per week) on any of the first 5 items of the Paediatric Insomnia Severity Index (PISI) reported by the parent at screening. Ineligible participants are those who currently meet or have previously met the criteria for a MDD diagnosis as determined by parent report on the mood module of the Kiddie Schedule for Affective Disorders and Schizophrenia (KSADS-COMP) [26] at baseline.

### Intervention condition

Participants allocated to the intervention condition receive access to the child-adapted version of Sleep Ninja for 6 weeks. Sleep Ninja is an automated smartphone application co-designed with 12- to 16-year-olds and parents to deliver CBT-I. It is free and publicly available in Australia on devices using iOS and Android operating systems. The program includes six sequential modules (referred to as ‘training sessions’), a sleep tracking function, sleep scheduling based on required wake time and age-dependent sleep duration recommendations, personalised wind-down routines and reminders, a series of sleep tips, and general information about sleep. Each training session covers a core CBT-I strategy including: (a) sleep scheduling; (b) stimulus control strategies; (c) sleep hygiene for evenings and daytime; (d) cognitive therapy including how to deal with thoughts that can prevent sleep onset (e.g., repetitive worrying) (e) identification and planning for high-risk situations and diversions from sleep routine; and f) relapse prevention. Sessions are delivered through an automated chat-bot where the Ninja acts as a sleep coach. Sleep Ninja is designed to be used during the day and not at bedtime to prevent device use and blue light interfering with sleep. The child version of Sleep Ninja also includes a website for parents, designed in collaboration with parents. The website contains guidance to assist parents in supporting their child to use Sleep Ninja autonomously and additional resources including information on Sleep Ninja, case examples, frequently asked questions, and contact information for the Child Sleep team at the Black Dog Institute. Parents of participants allocated to receive the intervention will be provided with the URL for the website alongside instructions on how to download Sleep Ninja. Child participants allocated to the intervention condition will be instructed to complete the Sleep Ninja app content by undertaking one ‘training session’ and tracking their sleep for three nights per week (approximately 10 min in duration) for six weeks. One email and one Short Message Service (SMS) reminder will be sent to the parents of participants who fail to download the Sleep Ninja app within 7 days of randomisation. Participants who do not download the app will remain in the trial and be included in the intention-to-treat analyses.

### Control condition

Participants allocated to the active control condition will receive six digital psychoeducation flyers, delivered weekly, that are optimised for viewing on mobile devices. Psychoeducation is a recognized and well-received intervention for sleep problems and has shown modest positive effects on outcomes without incorporating active cognitive-behavioural therapy (CBT) components [27–29]. Our active control has also been matched to the intervention condition in terms of visual appeal, weekly content dosage, and engagement duration. This provides control participants with helpful but non-therapeutic information related to sleep, thereby minimizing the likelihood of dropout in the control condition [30]. The psychoeducation consists of six topics (taking approximately 10 min to read): (1) Why do we sleep? (2) Fun facts about sleep, (3) Tips to help you sleep-Part 1, (4) Tips to help you sleep-Part 2, (5) Stress and sleep, (6) Where to get more help. The content was created specifically for this trial and to be appropriate for 10 to 12 year olds. The content has a readability score at the Year 5 to 6 level (i.e., for readers aged 10 to 12). All participants in the control condition will be provided with access to Sleep Ninja once their participation in the trial has concluded (i.e., 9-months post-baseline).

#### Procedure and participant timeline

**Fig. 1:**
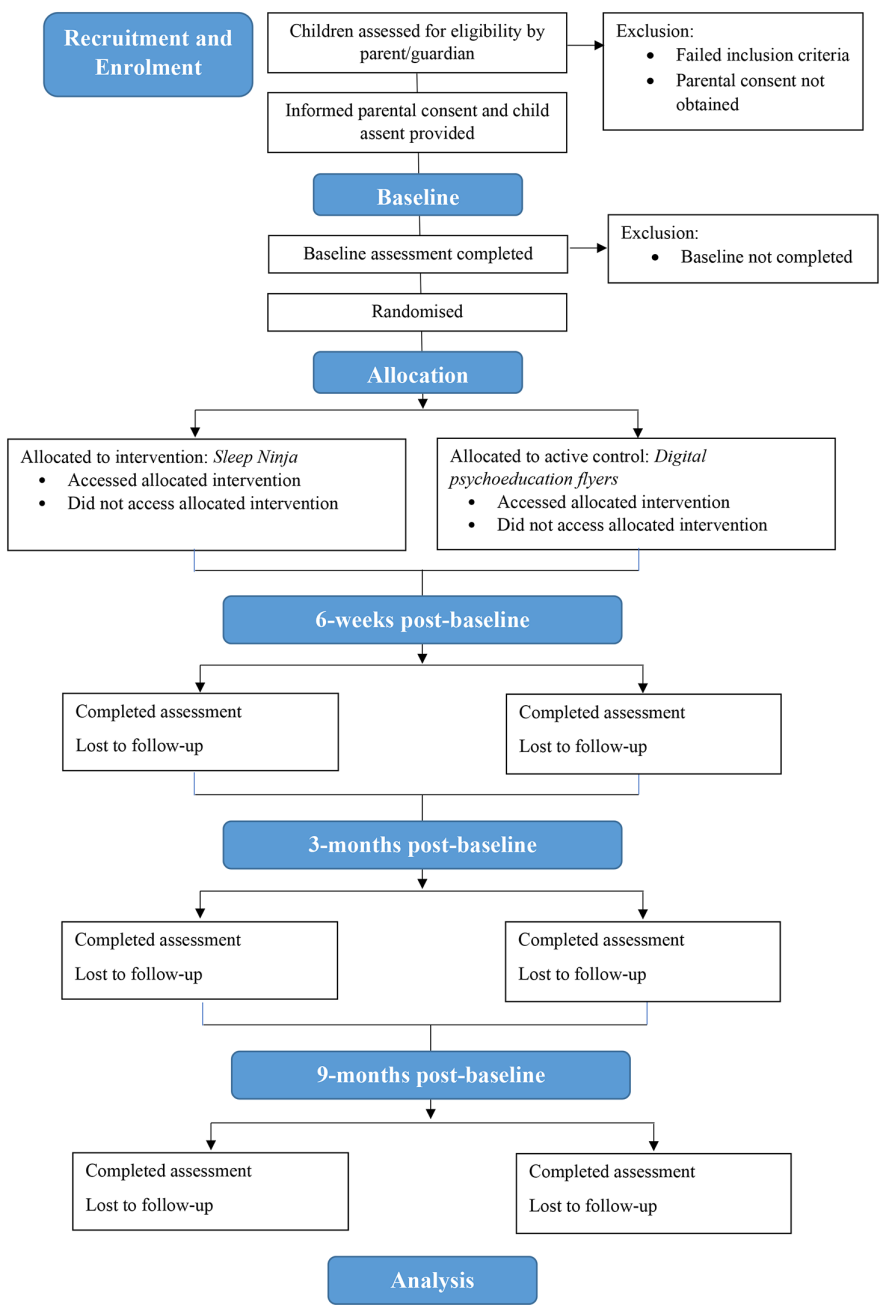
Participant recruitment and study flow diagram

Table 1 outlines the schedule of enrolment, interventions, and assessments. Figure 1 outlines the study flow.

**Table 1:** Schedule of enrolment, interventions, and assessments

TIMEPOINT	STUDY PERIOD					
	Screening	Baseline	Intervention period	Post intervention assessments		
			*Days 1–42*	*Primary endpoint (6-weeks post baseline)*	*Secondary endpoint (3-months post-baseline)*	*Tertiary endpoint (9-months post-baseline)*
**ENROLMENT**:						
Eligibility screening; Parent self-report	X					
Parental consent	X					
Personal Child assent	X					
Registration	X					
Randomisation		X				
**INTERVENTIONS**:						
1. Sleep Ninja®			↔			
2. Digital psychoeducation flyers via SMS (active control)			↔			
**ASSESSMENTS**:						
*Primary Outcome*						
**Sleep problems** Paediatric Insomnia Severity Index - Adolescent (PISI-A); Child self-report		X		X	X	X
*Secondary Outcomes*						
**Sleep problems** Paediatric Insomnia Severity Index (PISI); Parent self-report		X		X	X	X
**Depressive symptoms** The Revised Child Anxiety and Depression Scale – 25; Subscale (RCADS-25); Parent self-report		X		X	X	X
The Revised Child Anxiety and Depression Scale – 25; Depression Subscale (RCADS-25); Child self-report		X		X	X	X
**Anxiety Symptoms** The Revised Child Anxiety and Depression Scale − 25; Anxiety Subscale (RCADS-25); Parent self-report		X		X	X	X
The Revised Child Anxiety and Depression Scale – 25; Anxiety Subscale (RCADS-25); Child self-report		X		X	X	X
**Daytime Sleepiness** The Epworth Sleepiness Scale; Child self-report		X		X	X	X
**Sleep Quality** Sleep Quality Question; Child self-report		X		X	X	X
**Dysfunctional beliefs about sleep** The Dysfunctional Beliefs and Attitudes about sleep (DBAS); Child self-report		X		X	X	X
**Pre-sleep hyperarousal and anxiety** The Sleep Anticipatory Anxiety Scale (SAAQ) Adolescent Version; Child self-report		X		X	X	X
**Repetitive Negative Thinking** The Persistent and Intrusive Negative Thoughts Scale (PINTS); Child self-report		X		X	X	X
**Quality of Life** The Child Health Utility 9D (CHU-9D); Child self-report		X		X		
**MDD Diagnosis** Kiddie Schedule for Affective Disorders and Schizophrenia (K-SADS-COMP; assessment of Mood disorders only); Parent self-report		X				X
*Additional study measures*						
**Registration details** Registration Questionnaire; Parent self-report		X				
**Demographics** Demographics Questionnaire; Parent self-report		X				
**Recent mental health care** Mental Health Care Questionnaire; Parent self-report		X		X	X	X
**Previous use of mobile apps for sleep** Mobile app questionnaire; Parent self-report		X				
**Digital program satisfaction** Satisfaction Questionnaire; Child self-report				X		
Treatment Adherence* Treatment adherence; Parent self-report				X		
**Withdrawal reasons** Withdrawal question		X	X	X	X	X

*Note.* *Assessed in the intervention group only

Parents who would like their child to take part will be directed to the study webpage and will be asked to complete a brief online screening questionnaire on behalf of their child to determine eligibility. Parents of children who do not pass screening will be provided with sleep health information and mental health service information. Only parents of eligible children will be invited to review the online Participant Information Statement and Consent Form (PISCF). Once parental consent is provided, a child-friendly version of the PISCF will be presented with the child’s assent assumed if they progress through and are registered for their trial by their parent. Parents of participants will be asked to register by providing the name of the parent and child, the parent’s email address, and the parent’s mobile phone number. This process has been successfully used in previous trials conducted online [17, 31].

Following registration, the parent and child will be directed to the first (baseline) study assessment, with two reminders sent if not completed (Days 3 and 5). Participants and their parents who fail to complete the baseline assessment within the allocated time will be automatically withdrawn from the study. Upon completion of the baseline assessment, participants will be randomised to one of the two study conditions. Parents of participants will receive an email containing information regarding their child’s allocated intervention (Sleep Ninja or digital psychoeducation flyers). At six-weeks post-baseline, 3-months and 9-months follow-up, all randomised participants will be invited via email and SMS to complete the study assessments.

Each study assessment contains questionnaires in relation to the child participant, with the baseline and 9-month follow-up assessment also containing a diagnostic assessment. Some questionnaires are to be completed by the parent (parent-report) and other questionnaires are to be completed by the child (child-report). Participants will have 14 days to complete the baseline assessment and 7 days to complete all other study assessments and will be sent two reminders (Days 3 and 5). All study assessments can be completed using any Internet-enabled device and include both parent- and child-reported measures. The research team will be available for contact throughout the trial period to participants in case assistance is needed, with research team contact details being provided on the study webpage, the Participant Information Sheet, and at each assessment. However, contact with participants will not be proactive or therapeutic in nature. Participants will be reimbursed $10AUD (electronic gift voucher sent to parents via email) for each study assessment and diagnostic assessment ($60AUD total). Discontinuation of the allocated intervention will occur if the participant withdraws from the trial or if they are no longer able to engage in the intervention due to a change in circumstances (e.g., reside outside Australia).

### Primary outcome

#### Insomnia symptoms (child-reported)

The primary outcome will be child reported insomnia, using the Paediatric Insomnia Severity Index – Adolescent (PISI-A). This measure is adapted from the original Paediatric Insomnia Severity Index, completed by parents. The measure contains 6-items. Items 1 to 5 assess the frequency of insomnia-related symptoms including difficulty falling asleep, nighttime wakings, and daytime sleepiness, which are all rated on a 6-point Likert scale from Never − 0 nights (0) to Always − 7 nights (5). Total sleep duration (item 6) is rated on a 6-point scale estimating total hours of sleep on most nights, where a lower score indicates more hours of sleep (0 = 11–13 h; 5 = less than 5 h). This measure has good validity and reliability in children aged 11–18 years [32–34]. The selection of a child-rated measure, over a parent-report measure was based on data showing parents of children aged 10–12 years are often unaware of their child’s sleep difficulties [11].

### Secondary outcomes

#### Insomnia symptoms (parent-reported)

The Paediatric Insomnia Severity Index (PISI) is a parent-reported measure of insomnia severity, which assesses the International Classification of Sleep Disorders (ICSD-II) general criteria for insomnia (i.e., difficulties falling asleep, difficulties maintaining sleep, and daytime impairment). The measure contains 6-items. Items 1 to 5 are rated on a 6-point Likert scale from Never − 0 nights (0) to Always − 7 nights (5). Total sleep duration (item 6) is rated on a 6-point scale estimating total hours of sleep on most nights, where a lower score indicates more hours of sleep (0 = 11–13 h; 5 = less than 5 h). The total score will be used (range: 0–30). The PISI has adequate validity and reliability in children aged 4 to 10 years [32, 33, 35]. As the child-reported sleep measure (PISI-A) is validated for children aged 11 years and above, we chose to include a parent-reported measure of child insomnia to ensure a validated measure of insomnia is included for participants aged 10 years old.

#### Depressive symptoms

Parent- and child-reported depressive symptoms will be measured by the depression subscale of the Revised Children’s Anxiety and Depression Scale-25 (RCADS-25) Parent Report and the RCADS-25 Child Report [36]. The depression subscale measures symptoms of depression in children and adolescents aged 8 to 18 years. Ten items assess the frequency of symptoms and are rated on a 4-point Likert scale from Never (0) to Always (3). The total score will range from 0 to 30, with higher scores indicating higher severity of depressive symptoms. Both the parent and child versions have good psychometric properties [37] and are consistent with current guidelines for the assessment of mental health in children [38].

#### Anxiety symptoms

Parent- and child-reported anxiety symptoms will be measured by the anxiety subscale of the Revised Children’s Anxiety and Depression Scale-25 (RCADS-25) and the RCADS-25 Child Report [36]. Fifteen items assess anxiety symptoms as rated by the parent and child and a rated on a 4-point Likert scale from Never (0) to Always (3), with total scores ranging from 0 to 45. Higher scores indicate higher severity of anxiety symptoms. The Total Anxiety subscale has been found to have good to excellent internal consistency and good to excellent reliability in school and clinical samples [37, 39].

#### Daytime sleepiness

The Epworth Sleepiness Scale (ESS) is a short questionnaire designed to assess daytime sleepiness in eight different situations, and a modified version has been designed for children [40]. Children are asked to rate on a 4-point scale (0 to 3), their usual chances of dozing off or falling asleep while engaged in eight different activities. The ESS total score can range from 0 to 24. The higher the ESS score, the higher that person’s average sleep propensity in daily life, or their ‘daytime sleepiness’. The ESS has demonstrated high internal reliability [41].

#### Sleep quality

As a validated child-report measure of sleep quality is not available, a single item on sleep quality was adapted for children from the Pittsburgh Sleep Quality Index (PSQI) specifically for this study. The wording of the adaptation was developed by consensus across three psychologists who specialise in sleep and tested in children aged 10 to 12 years old (N = 5). The question asks children to: ‘Rate how well you have been sleeping over the last month’, with responses ranging from ‘Very well’ (0) to ‘Not at all well’ (3).

### Measures of mechanisms

All potential mechanisms will be measured at all four assessments (baseline, primary, secondary, and tertiary endpoints) and explored as mediators of change.

#### Dysfunctional beliefs about sleep

To determine the effect of the intervention on dysfunctional beliefs about sleep and explore its role as a potential mediator, The Dysfunctional Beliefs and Attitudes about Sleep for Children (DBAS-C10) questionnaire [42] will be used. It contains 10-child-reported items measuring dysfunctional beliefs about sleep. Items are rated on a Likert scale from (1) Strongly Disagree to (5) Strongly agree [42]. The DBAS-C10 has been adapted for use with children and has demonstrated validity and reliability [43].

#### Pre-sleep arousal, and pre-sleep cognitions

The Sleep Anticipatory Anxiety Scale – Adolescent Version (SAAQ) [44] is a child-reported measure of anxiety levels before going to bed or during the pre-sleep period. The SAAQ assesses the intensity of anxiety symptoms related to sleep, including worry, restlessness, and anticipatory thoughts via three subscales: Somatic, Sleep-related Cognitions and Rehearsal/Planning. Each item is scored on a 4-point Likert scale ranging from Strongly Disagree (1) to Strongly Agree (4). The scale measures the degree of anxiety individuals may experience in relation to sleep and is widely used in youth samples with demonstrated validity and reliability [45].

#### Repetitive negative thinking

The Persistent and Intrusive Negative Thoughts Scale (PINTS) [46] is a 5-item questionnaire that assesses the frequency and intensity of negative thoughts that may contribute to emotional distress in children, adolescents, and adults. The PINTS has demonstrated good internal consistency and reliability in children [47].

### Exploratory outcomes

#### Major depressive disorder

The web-based Kiddie Schedule for Affective Disorders and Schizophrenia (K-SADS-COMP; assessment of mood disorders only) [26] is a commonly used parent-report tool used to assess Diagnostic and Statistical Manual of Mental Disorders-5 (DSM-5) diagnostic criteria [47]. The mood disorder module for childhood psychiatric diagnoses [26] will be used to assess the presence of current at past MDD. While the instrument comprises 22 diagnostic modules, only the assessment of mood disorders will be included in this study taking less than 30 min to complete. It will be administered at baseline and at 9-months post-baseline.

#### Quality of life

The Child Health Utility 9D (CHU-9D) [48] assesses adolescents’ health-related quality of life in nine domains of functioning including worry, sadness, pain, tiredness, annoyance, schoolwork/homework, sleep, daily routine, and ability to join activities. It contains 9-items, with each domain rated on a 5-point scale and higher scores indicating greater severity (e.g., I don’t feel worried today (1) to I feel very worried today (5)). Items are summed to produce a total score ranging from 9 to 45. The scale has good psychometric properties among children (7–17 years) [49] and can estimate quality adjusted life years for use in future cost-effectiveness evaluations of Sleep Ninja. It will be administered at baseline and at 6-weeks post-baseline.

### Other measures

#### Demographic information

At baseline, parents of participants will be asked their child’s date of birth, school grade (Year 4, 5, 6), gender identity (female, male, non-binary, different identity, free response option), whether they identify as Aboriginal or Torres Strait Islander (yes, no, prefer not to say), their Australian state or territory location (NSW, QLD, VIC, TAS, SA, WA, NT, ACT), location description (metropolitan, regional, or rural/remote), and the type of mobile device they use (iOS [e.g., Apple], Android [e.g., Samsung], other). Participants will also be asked how they heard about the study (e.g., social media advertisement, Black Dog Institute website, word of mouth, other) and their motivations for participating (e.g., need for mental health care, desire to contribute to a broader social good, parents/carers encouragement, friend participating, financially motivated, desire to help research, interest in learning about mental health or other).

#### Recent mental health care

At baseline, parents of participants will be asked to report whether their child has ever been diagnosed with insomnia or another mental illness by a health professional (yes – no, I don’t know, I’d rather not say), with an open response option to list the ‘other’ mental illnesses, whether their child has received psychological treatment for insomnia by a mental health professional (yes, no, I’d rather not say) and whether their child has ever taken prescribed or over the counter medication for insomnia (yes, no, I’d rather not say). At the primary, secondary, and tertiary endpoints, parents will be asked the same questions to determine whether throughout the study their child received a diagnosis of insomnia, received psychological treatment from a health professional, started taking medication (prescribed or over-the-counter) for insomnia or felt their child needed mental health support. They will also be asked if their child received a diagnosis of depression or anxiety since the last assessment.

#### Previous use of mobile apps for sleep and expectancies

At baseline, parents will be asked to report whether their child has ever used a mobile app to assist with their sleep (yes, no, I’d rather not say), and how useful they think a mobile app for sleep would be for their child (not at all useful, somewhat useful, neutral, very useful, extremely useful). Parents will also be asked how much they think their child’s sleep or emotional well-being could be improved by using a mental health smartphone app.

#### Digital program satisfaction

At the primary endpoint, satisfaction with the app in the intervention group and satisfaction with the digital psychoeducation flyers in the control group will be assessed using an 11-item measure adapted from previous digital research [50]. This measure is completed by the child and will assess their satisfaction, experience, and perceived helpfulness of the intervention. The first eight items assess participants’ satisfaction and experience with the Sleep Ninja app by asking participants to indicate their agreement or disagreement with each statement. Additionally, participants are asked to rate the overall helpfulness of the intervention on a 5-point scale, ranging from Extremely Unhelpful (1) to Extremely Helpful (5). The final two free response questions examine how Sleep Ninja, or the digital psychoeducation flyers were helpful (e.g., In what ways did Sleep Ninja help you?), and another giving participants the opportunity to provide suggestions for improvement (e.g., What would make Sleep Ninja better?).

#### Intervention adherence and attrition

At the primary endpoint, treatment adherence (to Sleep Ninja) will be assessed using an 11-item, parent-report measure in the intervention group only. The first five items are rated on a 7-point Likert scale from Strongly Disagree (1) to Strongly Agree (7) and assess the child’s use (e.g., My child used Sleep Ninja more than once a week) and changes to behaviour (e.g., My child went to bed at the same time at night and woke at the same time in the mornings). Item 6 asks how many training sessions were completed by the child, with the option to select from 1 to 6. The remaining items assess the degree of parent involvement in the intervention, including frequency and duration of involvement, visits to the parent website, and a free response item allowing parents to describe how they supported their child’s use of the Sleep Ninja app. Data automatically collected within the app will also be used to provide objective measures of app usage, such as whether the app was downloaded, the number of sessions completed, and total time spent using the app. The number of users interacting with the parent webpage and the number of visits per user will be obtained from Google Analytics 4. For participants allocated to the control condition, access to the psychoeducation flyers will be recorded for each participant via URL clicks. Attrition is operationalised as non-completion of the outcome measures and will be reported for each assessment occasion.

### Sample size

The total sample size required for detecting change in the primary outcome at the primary endpoint was calculated to be 214. This was based on using α = 0.05, power = 0.8 to detect a medium between-group effect size (d = 0.41) [17, 22] on child-reported insomnia outcomes. This estimate accounts for an attrition rate of 40% between baseline and the primary endpoint. The effect size parameter used in the power calculation accounts for potential effects of the active control condition, as seen in the adolescent study [17], but is more conservative than the between-group effect size reported in that study (d = 0.41) due to the differences in the delivery format (parent-supported vs. self-directed).

### Recruitment and screening

Trial recruitment will begin in August 2023 and will continue until the required sample size is achieved. This study will utilise an online recruitment strategy targeting parents/guardians of children aged 10 to 12 years as this recruitment method has been established to recruit parents and young people [22]. Study advertisements will be published on the Black Dog Institute’s website and social media channels (Facebook, Instagram, and Twitter) and through Institute communications via established professional newsletters. Paid advertisements will be utilised on social media sites targeting parents (Facebook, Instagram, Twitter, LinkedIn) and Google. The research team will also send two email invitations to adults who have consented to be contacted about research studies conducted at the Institute. Additionally, the research team will contact primary schools via email to share study information with their communities. Contact will also be made by the research team to relevant Australian mental health organisations and services including psychology clinics and parent and youth groups to request distribution of study advertisements through these organisations’ communication channels (e.g., website, newsletters, social media, in-person clinics). Study advertisements will contain a URL directing all interested participants to the study’s webpage where they can review study information, and may choose to commence screening, and provide consent using the provided links. Direct contact between the participant and the study team is not required during recruitment and study enrolment. Parents will complete screening on behalf of the child participant. To ensure participant safety during recruitment and screening, parents of excluded participants will be provided with contact information for Australian mental health and crisis support services and encouraged to seek help if mental health support is required for either themselves or their child. Tips about healthy sleep will also be provided.

### Randomisation

Randomisation to one of the two trial conditions, stratified by gender (male, female, gender diverse) will occur immediately after completion of the baseline survey using a computerised randomisation procedure within the Qualtrics platform and will be carried out according to the International Council for Harmonisation guidelines [51]. Randomisation will be fully automatic, with no input from the research team.

### Blinding

Regarding participant blinding, all study materials will refer to the interventions being examined as “activities to improve sleep” to conceal Sleep Ninja being the smartphone app used in the trial. This is to prevent participants allocated to the control condition from accessing the Sleep Ninja smartphone app, which is publicly and freely available for download in Australia, during their participation in the trial. While participants will be aware of which activity or information they will be required to use (due to the PISCF and instructions for use provided), they will not be directly informed of their condition allocation (i.e., intervention vs. control) to prevent biases in reporting related to expectancies.

The statistician involved in examining the effects of the conditions of primary and secondary outcomes will not be informed of participants’ specific intervention allocation. Condition allocated will be marked as “Condition A, Condition B” to ensure the statistician remains blinded to participants’ intervention upon primary and secondary analysis of the results. All other potential markers of allocation will be removed from the data analysis file. Upon completion of these analyses, the statistician will be become unblinded when examining mediators and moderators as well as intervention completion rates. This data will only be reviewed by the trial statistician upon completion of the primary and secondary outcomes.

The Chief investigators, trial manager and research assistants will be unblinded to participants’ allocation as they will have access to the Qualtrics data to contact participants if they experience adverse events (see Monitoring below for more information). They will also be responsible for downloading and cleaning the data extracted. The Chief Investigators, Trial Managers, Data Analyst and Research Officers/Assistants will not be involved in data analysis of the primary and secondary outcomes.

### Data collection, management, and statistical methods

Participant data will be collected and securely stored on the University of New South Wales (UNSW) OneDrive which are hosted on servers provided by the UNSW at GovDC Data Centres in Sydney, Australia. The data is stored in a SQL Server 2016 database, which undergoes daily backups and is supported by 128-bit encryption. To ensure participant confidentiality, at the data analysis stage, each participant will be assigned and only identified by a unique identification code.

For analyses, all study data will be exported from the Qualtrics Survey Platform to SPSS Version 26 using a Microsoft Excel file format. Files with identifiable data will be stored on UNSW OneDrive and once deidentified by authorized members of the research team for analysis will be stored on a secure sever. Any personally identifiable information, such as participant names, email addresses, mobile phone numbers, IP addresses, free response data, will be removed from the outcomes analysis file. Only the research team listed on the project will have access to identifiable study assessment data, while aggregate deidentified data will be retained for future evidence synthesis.

Analyses will be conducted to determine the effect of the interventions on insomnia symptoms at the primary and secondary endpoints. Analyses will be undertaken on an intention-to-treat basis, including all participants randomised, regardless of the intervention received. The effectiveness of the trial intervention will be established using mixed-model repeated measures (MMRM) analyses due to the inclusion of participants with missing data with this approach. The primary hypothesis will be evaluated by a contrast evaluating change in child-reported insomnia symptoms between baseline and 6-weeks post-baseline (primary endpoint), based on the interaction between time and condition. The same approach will be used for secondary outcomes. Adherence and acceptability of the intervention will be described.

Exploratory analyses will compare proportion of young people who meet criteria for MDD at the tertiary endpoint, as well as those showing significant symptoms of depression, operationalised as a t-score of greater than 65 on the RCADS.

To determine whether improvements in depression are mediated by changes in sleep, an additional MMRM analyses will be conducted using depression symptoms as the outcome and including a 3-way interaction term (time × group × insomnia). Similarly, to explore other potential mediators of treatment effects, additional MMRM analyses will be used and include 3-way interaction terms (time × group × mediator).

A deidentified data set may be requested by emailing the study team. Release of the data will be decided on a case-by-case basis and at the discretion of the Chief Investigator and facilitated through a data repository using Research Data Australia.

### Monitoring

The current trial will be overseen by a Trial Management Group to supervise the conduct and safety of the trial in accordance with the guidelines on Data Safety Monitoring set by the National Health and Medical Research Council [52]. Parents of participants who report an Adverse Event (AE), which includes severe insomnia symptoms (indicated by a score of 5 on item 6 of the PISI) reported at the tertiary endpoint (9-months post-baseline), will receive a pop-up message in the questionnaire with recommendations to seek support from a health professional for their child. A call-back from a psychologist on the research team will also be offered. Participants who report an AE that includes severe depressive symptoms (indicated by a total t-score ≥ 70 on the RCADS) reported at the tertiary endpoint (9-months post-baseline) will receive a pop-up message in the questionnaire recommending that they tell a trusted adult about how they are feeling and seek help from a health professional. They will be provided with information about where to seek help and informed that we will let their parents know they are having a hard time. An email will be sent to the parent or guardian’s email address informing them of their child’s response with the option of a call-back from the team Psychologist. To examine rates of symptom deterioration, an increase of seven points on the PISI-A will be used as the threshold to indicate reliable symptom deterioration at the primary endpoint. This criterion is based on a α = 0.8 and a SD = 5.4 as recommended by Jacobson and Truax [53] and Byars and Simon [32]. All adverse events and serious adverse events and broader safety monitoring will be documented and reported to the Trial Management Group and to the University of New South Wales Human Research Ethics Committee by the Clinical Trial Manager and reported in the primary outcomes paper. If there are concerns for participant safety, based on (but not limited to) a higher than anticipated rate at one or more of the endpoints, the Trial Management Group may recommend pausing or terminating the trial. The University of New South Wales may audit trial conduct and procedures at any time and this process will be independent of the investigators and the sponsor.

### Dissemination

Upon completion of data analysis, a one-page summary of the research results will be made available on the Black Dog Institute website and will be emailed to all parents of participants. We will encourage parents to share the findings with their child. The research team will prepare the trial outcomes for publication in relevant peer-reviewed journals and present outcomes at scientific conferences. To comply with the National Health and Medical Research Council open access policy, the researchers will make efforts to publish all papers resulting from this research in open access journals. In all reports, participants will not be individually identifiable and numerical data will be presented in aggregate form. Any qualitative data reported will be represented with non-identifiable codes.

## Discussion

This protocol outlines a clinical trial that aims to examine the effect of a CBT-I smartphone app (Sleep Ninja) on insomnia symptoms and other mental health symptoms in children aged 10- to 12 years experiencing frequent sleep disturbance. To the best of our knowledge, this will be the first study to evaluate app-delivered CBT-I in children with sleep difficulties. Sleep Ninja was originally developed collaboratively with young people, to address a lack of digital, self-guided CBT-I programmes to improve adolescent sleep and mental health symptoms [22]. Sleep Ninja has been shown to improve sleep and depression in adolescents experiencing insomnia symptoms [17]. With high rates of insufficient and disturbed sleep occurring in children and rising rates of childhood depression [54], alongside limited access to appropriate care [55], access to evidence-based care is needed. If found to be effective, Sleep Ninja has the potential to provide an accessible, free, and scalable treatment to improve insomnia and possibly other mental health symptoms in children. The outcomes of this trial will contribute to our understanding of utilising digital technologies to support children experiencing sleep difficulties and inform innovations in the field.

### Strengths

This is the first randomised controlled trial to evaluate app delivered CBT-I in children under 12 years with frequent sleep disturbance. The intervention under evaluation provides a brief, self-guided and time efficient treatment, which is a treatment option for sleep not currently available for 10 to 12 year olds. The trial design includes an active control condition that allows evaluation of the effect of Sleep Ninja beyond non-therapeutic effects, such as knowledge gained from generic sleep information widely available online and parental engagement. The sample size (N = 214) will ensure adequate power to detect intervention effects on insomnia. Finally, this study improves on previous studies by assessing diagnostic criteria for MDD. Although base rates of depression in this age group are low [56, 57], using diagnostic criteria as assessed by the KSADS-COMP goes beyond symptom measures and ensures the sample in this study do not have a history of depression, thereby allowing exploration of the notion that treating sleep reduces risk of subsequent depression.

### Limitations

Given Sleep Ninja is publicly available, contamination bias may occur. To minimise the risk of contamination, Sleep Ninja is not named in any of the recruitment or trial information. The use of sleep applications is also being assessed at each assessment to monitor and identify the use of Sleep Ninja by participants in the control condition. Despite these limitations, the results of this trial will offer valuable insights into the potential of a CBT-I smartphone app to address a gap in the current treatment landscape and shed light on the factors that contribute to therapeutic benefits in children aged 10 to 12 years old.

### Electronic supplementary material

Below is the link to the electronic supplementary material.

Supplementary Material 1: SPIRIT Checklist

## Data Availability

Data sharing is not applicable to this article as no datasets were generated or analysed during the current study. A detailed study design and analysis plan were preregistered, including access to material, see https://www.anzctr.org.au/Trial/Registration/TrialReview.aspx?id=385818.

## List of Abbreviations

AE: Adverse Event
CBT: Cognitive Behavioural Therapy
CBT-I: Cognitive behavioural therapy for insomnia
ESS: Epworth Sleepiness Scale
DBAS: Dysfunctional Beliefs and Attitudes about Sleep
DSM: Diagnostic and Statistical Manual of Mental Disorders
ICSD: International Classification of Sleep Disorders
KSADS: Kiddie Schedule for Affective Disorders and Schizophrenia
MDD: Major Depressive Disorder
MMRM: Mixed-model repeated measures
NHMRC: National Health and Medical Research Council
PINTS: Persistent and Intrusive Negative Thoughts Scale
PISI: Paediatric Insomnia Severity Index
PISCF: Participant Information Statement and Consent Form
PSQI: Pittsburgh Sleep Quality Index
RCADS-25: Revised Children’s Anxiety and Depression Scale-25
RCT: Randomised Controlled Trial
SMS: Short Message Service
SAAQ: Sleep Anticipatory Anxiety Scale
UNSW: University of New South Wales
